# Supplementary material for: Prosociality predicts labor market success around the world
Source: Nat Commun. 2020 Oct 20;11:5298. doi: 10.1038/s41467-020-19007-1 (PMC7576798; doi:10.1038/s41467-020-19007-1)
Supplement: Supplementary file 1 — Supplementary Information [file 41467_2020_19007_MOESM1_ESM.pdf]

Supplementary Information

Prosociality predicts labor market success around the world

Fabian Kosse[a] and Michela M. Tincani[b]

[a]Ludwig-Maximilians-Universität München and Institute on Behavior and Inequality
[b]University College London and Institute for Fiscal Studies

Published in *Nature Communications*

	(1) Log HH income	(2) Underemployed	(3) Unemployed
Prosociality (standardized)	0.060***	-0.011***	-0.008**
	(0.009)	(0.004)	(0.003)
Cognitive ability (std.)	0.101***	-0.008*	-0.003
	(0.008)	(0.004)	(0.003)
Age (in years)	0.011***	-0.017***	-0.014***
	(0.002)	(0.001)	(0.001)
Age squared / 100	-0.018***	0.015***	0.013***
	(0.002)	(0.001)	(0.001)
Female dummy	-0.060***	0.064***	0.048***
	(0.013)	(0.009)	(0.009)
Observations	77,522	45,677	45,677

Supplementary Table 1: Coefficients are OLS estimates, standard errors (clustered at country level) are displayed in parentheses, observations are weighted by the sampling weights provided by Gallup to achieve (ex post) representativeness. All regressions include sub-national region fixed effects. HH means household. Data source: GPS and Gallup World Poll (76 countries). Significance levels regarding two-sided t-tests: * $p < 0.1$, ** $p < 0.05$, *** $p < 0.01$.

Variables	(1)	(2)	(3)	(4)	(5)	(6)	(7)
(1) Log HH income	1						
(2) Underemployed	-0.187	1.000					
(3) Unemployed	-0.127	0.688	1.000				
(4) Prosociality	0.089	-0.042	-0.032	1.000			
(5) Cognitive ability	0.218	-0.048	-0.024	0.177	1.000		
(6) Age (in years)	0.138	-0.175	-0.172	0.029	-0.052	1.000	
(7) Female dummy	-0.054	0.073	0.069	0.035	-0.011	0.005	1.000

Supplementary Table 2: Individual level pairwise Spearman correlation coefficients for all used variables. HH means household. Observations between 45,686 and 77,536. Data source: GPS and Gallup World Poll (76 countries).
